# Supplementary material for: Netcombin: An algorithm for constructing optimal phylogenetic network from rooted triplets
Source: PLoS One. 2020 Sep 18;15(9):e0227842. doi: 10.1371/journal.pone.0227842 (PMC7500971; doi:10.1371/journal.pone.0227842)
Supplement: S4 File — (ZIP) [file pone.0227842.s004.zip › SequenceData-Triplet/sequenceData-10-20-30-0-Triplet/length 200, numseq 30, rate 10^-7/4 seq.pdf]

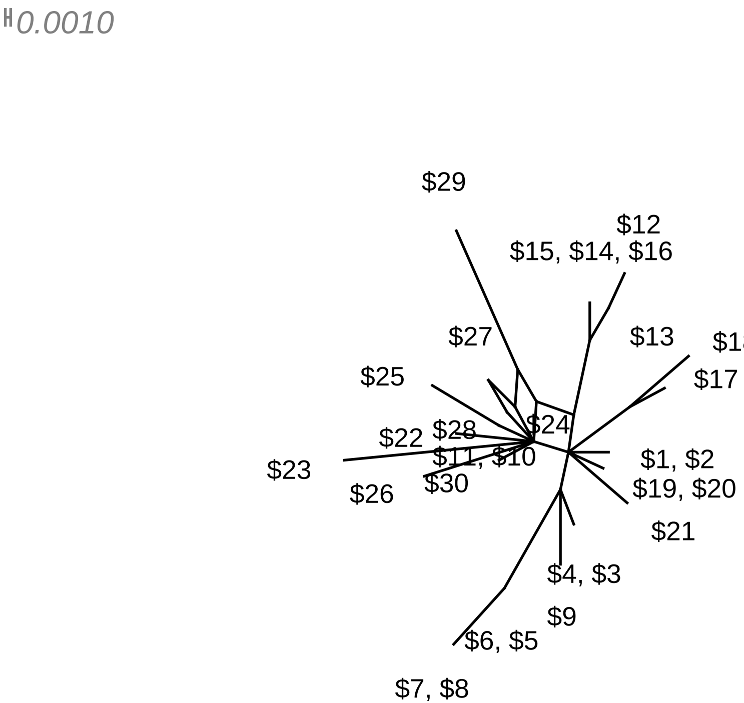
0.0010
$29
$12
$15, $14, $16
$27
$13
$18
$25
$17
$24
$22
$28
$11, $10
$23
$1, $2
$30
$19, $20
$26
$21
$4, $3
$9
$6, $5
$7, $8
